# Predictors of yearly influenza vaccination in hospitalized and community based patients

**DOI:** 10.1186/s40248-018-0135-6

**Published:** 2018-08-01

**Authors:** Stephanie Attard Camilleri, Juanita Camilleri Casingena, Kentaro Yamagata, Martin Balzan

**Affiliations:** 1 Health-Mater Dei Hospital, Msida, Malta; 2 Health-Primary Health Care, Bulawayo, Zimbabwe

**Keywords:** Immunisation, Influenza vaccine, Pneumococcal vaccine

## Abstract

**Background:**

Understanding positive and negative influences on adult immunization status can help healthcare providers to better identify and target patients who are likely to need immunization. Our aim was to assess and compare influenza and pneumococcal (IV/PV) immunisation rates to identify vaccination predictors in Malta.

**Method:**

One group consisted of all medical patients discharged from Mater Dei Hospital (MDH) over a one week period in February 2013. Patients were administered a phone questionnaire. A second group of patients receiving community-based care at local health centres over a one week period in March 2013 were interviewed, identifying vaccination eligibility as per 2010 WHO recommendations.

**Results:**

A total of 150 community (Mean age 61.5 SD 15.8, Male 60%) and 149 hospitalised (Mean age 66.8, SD 13.6%, Male 48.3%) patients in whom influenza vaccine was indicated were recruited. In the current year, 44 and 48.3% received the seasonal influenza vaccine, while 32.0, and 49% vaccinated yearly respectively. Pneumococcal vaccination advice was less than 5% in both groups. On stepwise binary regression, vaccination predictors for the current year were regular yearly influenza vaccination (OR 93.62, CI: 31.8–275.5, *p* < 0.001) and vaccination reminders (OR 27.5, CI: 9.63–78.31, p < 0.001). Nursing home residence (OR 5.78, CI: 1.22–27.4,*p* = 0.011), congestive cardiac failure (OR 2.11, CI: 1.1–4.08, *p* = 0.02) and diabetes mellitus (OR 1.68, CI: 1.04–2.72, *p* = 0.034) were all predictors for vaccination on exclusion of the strongest two predictors. For successive yearly vaccination, influenza vaccine recommendation by healthcare professionals (OR 12.35, CI: 4.5–33.91, *p* < 0.001) and vaccination reminders (OR 5.99, CI: 3.13–11.45, *p* < 0.01) were main predictors. Congestive cardiac failure (OR 2.37, CI: 1.20–4.7, *p* = 0.13) and nursing home residence (OR 7.07, CI: 1.45–34.5, p = 0,005) were also positive predictors. Male gender was a negative predictor (OR 0.51, CI: 0.31–0.83, *p* = 0.006). Some of those who did not vaccinate were unaware of such need (40.5% of community and 15.6% of hospitalised patients).

**Conclusions:**

Just under half of the patient population received the IV during 2012–2013 period. Hospitalized patients are more likely to vaccinate regularly while a large proportion of community patients are unaware of the indication to vaccinate.

**Electronic supplementary material:**

The online version of this article (10.1186/s40248-018-0135-6) contains supplementary material, which is available to authorized users.

## Background

The Advisory Committee on Immunization Practices (ACIP) and the World Health Organisation (WHO) 2012 recommendations advise yearly influenza vaccination as well as pneumococcal vaccination as shown in Tables 1 and 2 with mutual agreement towards vaccinating especially vulnerable groups such as the elderly, very young patients with chronic disease and the immunocompromised [1–6]. Evidence shows that influenza vaccination was associated with a lower admission rate from influenza complications, fewer deaths during the influenza season and decreased healthcare costs in the elderly and in the general population [7, 8]. There are two types of pneumococcal vaccines (PV): pneumococcal conjugate vaccine 13 valent (PCV13) and pneumococcal polysaccharide vaccine 23 valent (PPSV 23). The PCV 13 is now recommended to all adults over 65 years old in addition to the previous recommendation of vaccinating the elderly > 65 with PPSV23 [9]. The Capita trial showed that among older adults, PCV13 was effective in preventing vaccine-type pneumococcal, bacteremic, and nonbacteremic community-acquired pneumonia and vaccine-type invasive pneumococcal disease but not in preventing community-acquired pneumonia from any cause [10]. The most recent recommendation of administering the PCV13 to all older adults will be re-evaluated in 2018 by the ACIP [9].

**Table 1:** WHO and ACIP recommendations for influenza vaccination 2012

Influenza Vaccination	
WHO recommendations	ACIP recommendations
Pregnant women at any stage of pregnancy	Pregnant women and neonates
Children aged 6 months to 5 years	All persons more than 6 months who do not have contraindications
Elderly individuals > 65 years	Adults aged > 65 years
Individuals with chronic medical conditions	Adults with chronic medical conditions
Healthcare workers	Immunocompromised persons

**Table 2:** WHO and ACIP recommendations for pneumococcal vaccination 2012

Pneumococcal Vaccination	
WHO recommendations	ACIP recommendations
Healthy elderly (> 65 yrs. of age), particularly those living in institutions	All persons aged 65 years or more
Patients with chronic organ failure	
Heart, lung, liver or kidney disease, diabetes mellitus and alcoholism	Chronic heart/lung/liver/kidney disease, DM and alcoholism
Children > 2 yrs. old at high risk for disease (splenectomised children and sickle-cell disease)	Patients with leukaemia/lymphoma/multiple myeloma
Patients with immunodeficiencies particularly those with functional or anatomical asplenia	Congenital/acquired immunodeficiencies, congenital/acquired asplenia; splenic dysfunction or splenectomy; organ transplantation or diseases requiring immunosuppressive drugs
Prevention of subsequent pneumococcal infection in patients recovering from proven or assumed pneumococcal pneumonia	Nephrotic syndrome
	HIV infection
	Others including: cochlear implants, CSF leaks and cigarette smoking

This study was performed in Malta, an island state forming part of the European Union (population c. 412, 655, (2013). The National Immunisation Schedule in Malta includes the influenza vaccine, which is recommended for children aged 6 months to 5 years, elderly over 55 years of age, patients with chronic diseases, healthcare related occupations, as well as those in contact with at risk groups [11]. The pneumococcal vaccine has not been included in the local immunisation schedule to date, however, physicians may prescribe it and patients can purchase it from the private pharmacies.

The aim of this study was to determine the local Influenza Vaccine (IV) and Pneumococcal Vaccine (PV) uptake rates for the primary and the secondary healthcare populations and to identify predictors of vaccination.

## Method

The study population consisted of patients in whom Influenza and/or pneumococcal Vaccination was indicated as per Table 1. A hospital group of adult patients (age > 18) who were discharged after acute medical care at Mater Dei Hospital (MDH) from 21^st^-28^th^ February 2013 from the department of medicine were contacted one week after discharge. MDH is the main University Hospital in Malta. The questionnaire was delivered via phone either in English or Maltese by 6 registered medical practitioners (Additional files 1 and 2, respectively). Questions 2 and 3 from the questionnaire identified patients eligible for vaccination and hence recruitement into the study. Their eligibility criteria were subsequently also confirmed by looking up information from discharge letters, cardiac laboratory database (for stress test, echocardiogram and coronary angiogram results) and the hospital laboratory database. A second group consisted of adult patients who received community-based healthcare services at three local public general practise health centres: Paola, Mosta and Floriana from 25^th^-31^st^ March 2013. The three largest community based general practices serving the island were interviewed by 3 registered medical practitioners using the same questionnaire. Patients were asked whether they had taken the IV and/ or PV (PCV 13 and/ or PPSV 23) and the reason for vaccinating or not. The questionnaire also included a number of questions which could potentially be used to identify predictors of vaccination for winter 2012–2013 and for yearly vaccination. Yearly vaccination was defined as vaccination for 2 consecutive years prior to the study period.

The data collected was inputted using Microsoft Office Access ® (Additional file 3) and analysed using Minitab 16 whilst binary logistic regression and stepwise regression were used to identify predictors of vaccination. A *p* less than 0.05 was taken to represent statistical significance. The Fisher Test was used to compare the two population characteristics and vaccine eligibility characteristics (Tables 3-5).

**Table 3:** Patient demographics and co-morbidities

Demographics	Community	Hosp Discharged	Total	*p*
	*n* = 150	*n* = 149	*n* = 299	
Mean age	61.5 +/− 15.80	66.5 +/−15.63		0.005
Males	*n* = 89 (59.3%)	*n* = 82 (50%)		0.097
Age Range	18–90	19–91		
Co-morbidities				
Age > 65	89 (59.3%)	100 (61%)	189	0.77
DM	50 (33.3%)	63 (38.4%)	113	0.35
IHD	23 (15.3%)	49 (29.9%)	72	0.002
CHF	12 (8.0%)	35 (21.3%)	47	0.001
CKD	11 (7.3%)	97 (59.5%)	108	< 0.0001
Lung disease	30 (20%)	28 (17.1%)	58	0.5
Liver disease	2 (1.3%)	5 (3%)	7	0.26
Alcohol abuse	3 (2.0%)	4 (2.4%)	7	1
Immune disease	1 (0.7%)	5 (3.1%)	6	0.22
Transplantation	1 (0.7%)	2 (1.2%)	3	1

**Table 4:** Pneumococcal and influenza vaccine eligibility, recommendations and uptake rates

	Community-based population	Hospital-based population	
	*n* = 150	*n* = 149	*p*
IV taken this year	66 (44.0%)	72 (48.3%)	0.42
IV taken yearly	48 (32.0%)	73 (49.0%)	0.003
PV advised	7 (4.7%)	6 (4.0%)	1
PV taken	3 (2.0%)	4 (2.4%)	0.79
Reminded this year	76 (51.3%)	81(54.4%)	0.56
IV recommended	97 (64.7%)	109 (73.2%)	0.13

**Table 5:** Influenza vaccination rates per eligibility criteria

	Hospital patient population (*n* = 149)	Community patient population (*n* = 150)	Total (*n* = 299)	*p*
	Take IV yearly	Take IV yearly		
Age > 65	53/100 (53.0%)	35/89 (39.3%)	88/189 (46.5%)	0.0794
DM	38/63 (60.3%)	15/50 (30.0%)	53/113 (46.9%)	0.0022
IHD	27/49 (55.1%)	8/23 (34.8%)	35/72 (48.6%)	0.133
CHF	23/35 (65.7%)	5/12 (41.7%)	28/47 (59.6)	0.182
CKD	46/97 (47.4%)	5/11 (45.5%)	51/110 (46.4%)	1
Lung disease	12/28 (42.9%)	10/30 (33.3%)	22/58 (37.9)	0.58
Resident in elderly home	8/8 (100%)	2/4 (50%)	10/12 (83.3)	0.09
Others*	10/21 (47.6%)	6/15 (40%)	16/36 (44.4%)	0.65

*Liver disease, organ transplantation, disorders of immune system, pregnancy, disability, alcohol abuse, health-related occupation

## Results

A total of 150 patients (60% male; mean age 61.5, SD 15.8) receiving community based healthcare services and 149 patients (50% male; mean age 66.5; SD 15.63) receiving MDH based healthcare services (60.5% of total discharges, 14.4% of discharged patients were excluded due to non-eligibility for either vaccine whilst the remaining 25.1% of discharged patients could not be reached), were interviewed. Table 3 shows the characteristics and co-morbidities of these two population groups. Table 4 shows responses to questions related to vaccination whilst. Table 5 shows the univariate comparison of the two groups for influenza vaccination rates for each of the eligibility criteria.

**Fig. 1:**
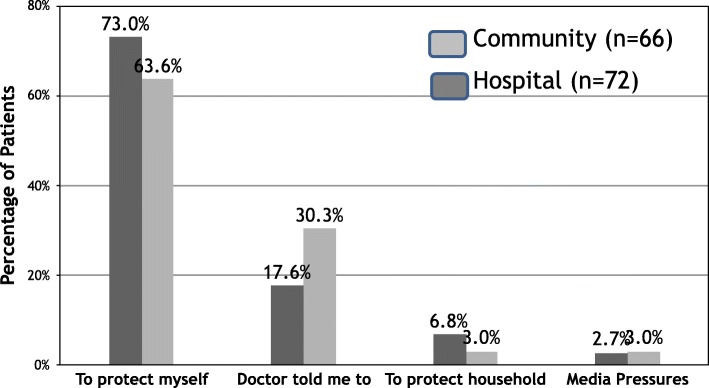
Reasons given by patients for taking the influenza vaccine for the winter 2012–2013

138(46.2%) patients (72, 48.3% MDH group; 66, 44% community group) took the influenza vaccine during winter 2012–2013. Fig. 1 (Reasons given by patients for taking the influenza vaccine for the winter 2012–2013) and 2 (Reasons given by patients for not taking the influenza vaccine for the winter 2012–2013) show reasons given by the patients for vaccinating or not vaccinating themselves during that winter for the MDH and the Community group of patients separately. Tables 6-8 show the binary logistic regression analysis with a stepwise selection model at *p* = 0.05 used to identify predictors of yearly influenza vaccination and predictors for vaccination for the year 2012–2013.

**Table 6:** Binary logistic regression model using all predictors (winter 2012/13)

Source	*P*		95% CI
Model	0.005	Odds Ratio	
Diabetes Mellitus	0.017	1.823	1.111–2.99
Male Gender	0.048	0.616	0.381–0.998
Chronic Heart Failure	0.058	1.974	0.972–4.009
Chronic Renal Failure	0.083	1.727	0.928–3.215
MDH/Community	0.125	1.561	0.879–2.774
Alcohol abuse	0.217	0.288	0.032–2.62
Age ≥ 65	0.26	1.01	0.993–1.027
Liver disease	0.413	0.491	0.085–2.846
Lung disease	0.75	1.106	0.595–2.056
Ischaemic Heart Disease	0.944	1.022	0.559–1.869

**Table 7:** Binary logistic regression to determine predictors for successive yearly vaccination

	Odds Ratio	95% CI	*p*
Male Gender	0.46	0.26–0.80	*p* < 0.001
IV recommendation	12.38	4.62–33.16	*p* < 0.001
Reminder to take IV	4.88	2.64–9.02	*p* < 0.01
On removal of the 2 strongest predictors			
Male Gender	0.48	0.29–0.77	0.02
Diabetes	1.65	1.01–2.69	0.047
CHF Y/N	2.80	1.44–5.44	0.002
Resident elderly home	7.49	1.55–36.17	0.004

**Table 8:** Binary logistics regression to determine predictors for vaccination for the winter 2012/13

	Odds Ratio	95% CI	*p*
Yearly IV vaccination	99.38	34.21–288.70	0.0001
Reminder to take IV	21.81	8.009–59.39	0.0001
On removal of the 2 strongest predictors			
Diabetes Mellitus	1.804	1.12–2.90	0.015
CHF Y/N	2.138	1.12–4.10	0.02
Resident in nursing home	6.455	1.37–30.37	0.006

## Discussion

This study aimed to investigate vaccination in a hospital based setting after discharge and in the primary care setting during the consultation of eligible patients. Patients’ eligibility was based on the WHO and ACIP criteria [2–6]. The hospital group was around 5 years older on average while the community group had a higher proportion of males. Cardiac and renal co-morbidities were higher in the hospital group.

Numerous studies have been performed attempting to identify factors which influence influenza vaccine uptake rates. However, most of the previous research focuses on factors which influence vaccination in the same year for a specific subset of patients [12–16]. This study not only aimed to identify predictors of vaccination for the winter 2012–2013 for two distinct patient groups: hospital and community but also to identify predictors for yearly vaccination. Only one hospital was included, but this is the only acute hospital in Malta and therefore it is inclusive and representative of the whole population. Patients from the community included the 3 main general practice health centres in Malta with the largest catchment areas, and the only 3 health centres which are open 24 h a day. Whilst not a random sample, the study population broadly represented users of the public health care system.

On the other hand, the main weakness of the study was the difference in conducting the questionnaire between the 2 population groups. This was done face to face in the community and via telephone in the hospital population group. This could possibly have introduced a small degree of bias. Another limitation of the study was that the number of people with alcohol problems, immune disease and organ transplantation was too small. Undoubtedly local factors due to the culture and delivery of the health service probably had a significant impact on the results possibly limiting the generalizability of the results.

At the time of the study, pneumococcal vaccination in Malta was low and the main limiting factor for vaccination in both groups was physician recommendation. Seven (out of 150) patients from the primary health care setting and 6 (out of 149) patients from the secondary healthcare group were told to take the pneumococcal vaccine at some point in their life. From these patients, only 3 from the community-based population and 4 from the MDH-based population actually took the vaccine. Lack of physician awareness on the importance of pneumococcal vaccination was the leading cause of low uptake rates in Malta. Another possible cause could be the fact that this vaccine has to be purchased by the patient as opposed to the free provision of influenza vaccine producing a financial barrier. Furthermore, since values for pneumococcal vaccine recommendation and uptake rates were too small, it was not possible to identify predictors of vaccination.

**Fig. 2:**
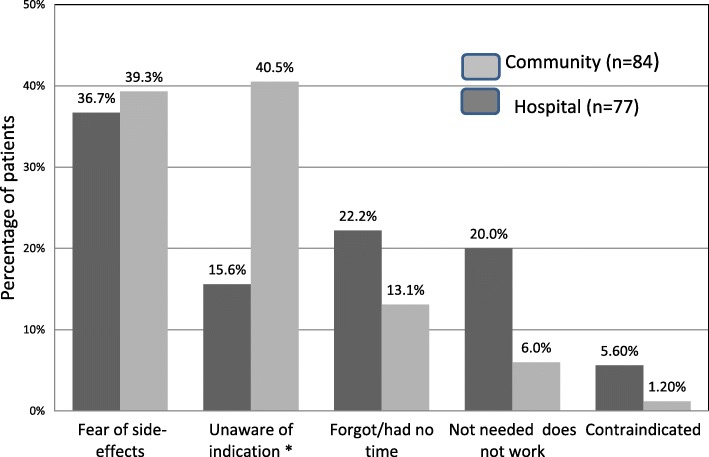
Reasons given by patients for not taking the influenza vaccine for the winter 2012–2013. * Statistically significant difference between MDH and Community population (*p* = 0.0003)

IV uptake, on the other hand, is comparatively higher than pneumococcal vaccination for both populations. Forty-nine per cent of the hospital based population and 32% of the community based population routinely take the IV every year with a significant difference between the two populations (*p* = 0.003). The data showed that hospitalised patients had more renal and cardiac co-morbidities than those receiving community-based care. Furthermore, the presence of an acute episode requiring hospitalization could indicate a more severe form of disease, which in turn could generate fear of future episodes and hence a stronger will to vaccinate regularly. This is supported by the fact that 73% of the MDH population stated that they vaccinated to protect themselves compared to 63.6% from the community based population. Logistic regression in Table 7 identified cardiac disease, nursing home residence, and hospitalization as independent predictors for yearly vaccination. Unfortunately 40.5% of community patients who did not vaccinate were unaware of the indication (Fig. 2: Reasons given by patients for not taking the influenza vaccine for the winter 2012–2013).

Table 7 showed that male gender was a negative predictor for successive yearly vaccination. The effect of gender on vaccination rates in the general population varies from country to country. A previous study of influenza vaccine uptake has also shown that female gender was one of the best predictors of vaccination [16]. This study might reinforce a possibility that women could be more aware of influenza risk and the necessity to vaccinate themselves.

For the current year 2012–2013, the strongest predictors for vaccination were a history of yearly influenza vaccination and having received reminders to vaccinate (Table 8). This data supports other studies where the need for vaccination through physician recommendation and yearly reminders to vaccinate, represent potentially effective ways to improve uptake rate [16]. When the effect of physician recommendation was removed from the statistical model, having Diabetes Mellitus or Congestive Heart Failure and being a resident in an elderly home became statistically significant predictors for current year vaccination. One possible reason for this is that DM is a chronic disabling disease and these patients tend to be reviewed regularly at the diabetic clinic whilst patients with CHF tend to have acute life-threatening exacerbations. Thus, both these conditions carry a heavy disease burden possibly leading to more frequent contact with their caring physician. In Malta, it is common practice that patients residing in elderly homes are offered and given the influenza vaccine on a yearly basis in their residence thus IV is easily accessible. This could explain why living in an elderly home emerged as a predictor for influenza vaccination despite having few patients with this criterion in our study. Table 6 shows that having Chronic Kidney Disease just fails to be a statistically significant predictor for vaccination (*p* = 0.083) and was in fact eliminated by stepwise regression from the model. On the other hand, surprisingly, having chronic lung disease did not predict vaccination possibly either because respiratory disease might be milder and more intermittent or because a large proportion of our patient population are cared for by non-respiratory physicians or GPs.

The main reasons given by patients for taking the IV during the winter 2012–2013, were to protect themselves from serious disease (73% for the hospital patient group; 63.6% for the community patient group) followed by physician recommendation (17.6% hospital patient group; 30.3% community patient group). This compared well with results documented in both local studies [16] as well as published studies done abroad [17, 18]. The fact that physician recommendation was the second commonest reason for vaccination given by the patients themselves was confirmed on multivariate analysis of the questionnaire.

Reasons for not vaccinating are similar between the primary and secondary care in our study, main reason being fear of side effects with 39.3% in the primary and 36.7% in the secondary setting. This is also similar to the other Maltese study with 43% giving this reason [8]. Other reasons not to vaccinate in our study were unaware of indication (15.6% MDH and 40.5% community) forgot/ had no time (22.1% MDH and 13.1% community), feel they do not need to take it or that it doesn’t work (20% MDH and 6% community) and history of contraindication to vaccinate (5.6% MDH and 1.2% community). In the study done by Thomas et al., 40.3% were not expecting to catch influenza and therefore thought it was unnecessary [17].

Increasing physician awareness and education on the importance of influenza vaccination would be expected to improve vaccine uptake rates through recommendation at each doctor-patient encounter.

## Conclusion

The study examined vaccination rates for a group of medical patients recently discharged from an acute hospital and another group of patients receiving community-based care. Pneumococcal vaccination was low and the main limiting factor was lack of physician recommendation. Hospitalized patients were older, and had more cardiac and renal co-morbidities. Influenza vaccination for the current year was the same in both groups, however, significantly more hospitalized patients vaccinated against influenza every year. This might be attributed to more frequent co-morbidity, more severe disease with possibly a stronger will to protect oneself. Predictors for yearly influenza vaccination included vaccine recommendation by healthcare professionals, reminding patients to vaccinate, residence in a nursing home, diabetes mellitus, congestive cardiac failure and female gender.

## Additional files

Additional file 1: Study questionnaire in english. (DOCX 87 kb)

Additional file 2: Study questionnaire in maltese. (DOCX 87 kb)

Additional file 3: Study dataset. (XLSX 55 kb)

## Abbreviations

ACIP: Advisory Committee on Immunization Practices
CHF: Congestive heart failure
CI: Confidence intervals
CKD: Chronic kidney disease
DM: Diabetes Mellitus
GP: General Practitioner
IV: Influenza vaccine
MDH: Mater Dei Hospital
OR: Odds ratio
PCV13: Pneumococcal Conjugate Vaccine 13 valent
PPSV23: Pneumococcal Polysaccharide Vaccine 23 valent
PV: Pneumococcal vaccine
SD: Standard deviation
WHO: World Health Organisation
