# Supplementary material for: Predictors of yearly influenza vaccination in hospitalized and community based patients
Source: Multidiscip Respir Med. 2018 Aug 1;13:23. doi: 10.1186/s40248-018-0135-6 (PMC6069836; doi:10.1186/s40248-018-0135-6)
Supplement: Supplementary file 1 — Study questionnaire in english. (DOCX 87 kb) [file 40248_2018_135_MOESM1_ESM.docx]

**GENERAL INFORMATION AND INCLUSION CRITERIA**

**Drug History on Discharge Letter**

**Are you compliant?**

- Almost always
- Sometimes
- Almost never

**1.1 : Gender**: Male Female

**1.2: Age:** ……….. years

**1.3: Year of Birth:** ………….

**2. Inclusion criteria for pneumococcal / influenza vaccine audit**

**2.1** Age > 65years old

**2.2** Diabetes (on diet / medications)

**2.3**  History of Myocardial Infarction / IHD

**2.3.1** EST positive

**2.3.2** Angiogram positive (intervention / medical Rx)

**Alcohol intake is excessive:**

Male: >4u / day

(28u wkly)

Female: >3u / day

(21u wkly)

**1 Unit of alcohol:**

½ pint of beer

½ glass of wine

1 shot of spirit

**2.4** Heart Failure / Pulmonary oedema

**2.4.1** Echocardiogram Abnormal -Specify………………………………………….

**2.5** Kidney Disease

**2.5.1** Latest eGFR …………… ( disease if <90)

**2.6** Liver Problems / Failure

**2.7** Hstory of alcohol Abuse (check reference table)

**2.8** Lung disease.

**2.8.1** Lung Function Tests: **2.8.1.1** FEV1…….. **2.8.1.2** FVC…….. **2.8.1.3** FEV1 / FVC ……..

**2.8.2** ABGs: **2.8.2.1** PH…….. **2.8.2.2** PCO2…….. **2.8.2.3** PO2…….. **2.8.2.4** HCO3……..

**2.9** History of Organ removal / transplantation. If yes, specify (spleen, lung etc.) ……………………………………………………………………………………………………………………………………………..

**2.10** Disorders of the immune system. If yes, specify

……………………………………………………………………………………………………………………………………………..

**3. Additional Inclusion criteria for influenza vaccine audit**

**3.1**  Are you pregnant? **3.1.1** If yes, how many weeks?....................wks

**3.2**  Resident in elderly home

**3.3** Health related occupation? **3.3.1** If yes, specify…………………………………………………....

**3.4** Disability? **3.4.1** If yes specify………………………………….…………………………………………….

**3.5** Are you in contact with people with above inclusion criteria

**AUDIT ON PNEUMOCOCCAL AND INFLUENZA VACCINES**

**4.1** Do you know if the pneumococcal vaccine is recommended for you? Yes No

**4.2** Have you ever been told to take the pneumococcal vaccine? Yes No

If yes, by whom: **4.2.1** Friends / Work Colleagues

**4.2.2** Nurse

**4.2.3** General Practitioner

**4.2.4**  Specialist doctor

**4.2.5** Media (TV/Radio/Newspaper/Magazine)

**4.3** Are you thinking about taking it? Yes No

**4.4** Where you offered the pneumococcal vaccine following discharge?

**4.4.1** Was it prescribed? Yes No

**4.5** Did you ever take the Pneumococcal Vaccine?

**If no, why?** You may choose more than one answer.

4.5.2.1 Fear of side-effects

4.5.2.2 I forgot / Had no time

4.5.2.3 I did not know I had to take it

4.5.2.4 Because it’s expensive

4.5.2.5 Media Pressures

4.5.2.6 Other……..………………………………………….....

**If yes, why?** You may choose more than one answer.

4.5.1.1 Because the doctor told me to

4.5.1.2 I decided to take it, to protect myself

4.5.1.3 To protect household contacts

4.5.1.4 Media Pressures (elderly, children etc)

4.5.1.5 Other……………….……………………………………………….....

**5.1** Do you know if the influenza vaccine is recommended to you? Yes No

**5.2** Have you ever taken the influenza vaccine? Yes No

**5.3** Do you take it every year? Yes No

**5.4** Have you been reminded this year to take it? Yes No

If yes, by whom: **5.4.1** Friends / Work Colleagues

**5.4.2** Nurse

**5.4.3** General Practitioner

**5.4.4** Specialist doctor

**5.4.5** Media (TV/Radio/Newspaper/Magazine)

**5.5** Have you taken the influenza vaccine this year?

**If no, why?** You may choose more than one answer.

**5.5.2.1** Fear of side-effects

**5.5.2.2** I forgot / Had no time

**5.5.2.3** I did not know I had to take it

**5.5.2.4** Because its expensive

**5.5.2.5** Media Pressures

**5.5.2.6** Other…………………………………………….....

**If yes, why?** You may choose more than one answer.

**5.5.1.1** Because the doctor told me to

**5.5.1.2** I decided to take it, to protect myself

**5.5.1.3** To protect household contacts

**5.5.1.4** Media Pressures (elderly, children etc)

**5.5.1.5** Other …………………………………………………………
