# Supplementary material for: Predictors of yearly influenza vaccination in hospitalized and community based patients
Source: Multidiscip Respir Med. 2018 Aug 1;13:23. doi: 10.1186/s40248-018-0135-6 (PMC6069836; doi:10.1186/s40248-018-0135-6)
Supplement: Supplementary file 2 — Study questionnaire in maltese. (DOCX 87 kb) [file 40248_2018_135_MOESM2_ESM.docx]

**Informazzjoni genarali u eligibilita ghat-tilqim**

**Medicini li tiehu**

**Tehodhom dejjem?**

- Kwazi dejjem
- Kultant
- Kwazi qatt

**1.1 : Sess**: M Ragel Mara

**1.2: Eta:** ……….. snin

**1.3: Data tat-twelid:** ………….

**2. Tilqim kontra pneumococcus/influenza rrakkomandata:**

**2.1** Eta > 65 snin

**2.2** Diabete (fuq dieta/medicini)

**2.3**  Tbati bi problem tac-cirkulazzjoni tal-qalb

**2.3.1** EST pozittiv

**2.3.2** Angiogram pozittiv (intervenzjoni /kura medica)

**Alcohol eccessiv:**

Ragel: >4u kuljum

(28u fil-gimgha)

Mara: >3u kuljum

(21u fil-gimgha)

**1 Unit alcohol:**

½ pinta birra

½ tazza inbid

1 shot ta’ spirti

**2.4** Qalb ghajjiena/ilma fil-pulmun

**2.4.1** Echocardiogram Abnormali -Specifika………………………………………….

**2.5** Mard tal-kliewi

**2.5.1** L-ahhar eGFR …………… (marid jekk <90)

**2.6** Mard fil-fwied

**2.7** Storja ta abbuz ta alcohol

**2.8** Mard fil-pulmun

**2.8.1** Lung Function Tests: **2.8.1.1** FEV1…….. **2.8.1.2** FVC…….. **2.8.1.3** FEV1 / FVC ……..

**2.8.2** ABGs: **2.8.2.1** PH…….. **2.8.2.2** PCO2…….. **2.8.2.3** PO2…….. **2.8.2.4** HCO3……..

**2.9** Storja ta trapjant/organi mnehhija. Jek iva, specifika liema organu (milsa, pulmun ecc.) ……………………………………………………………………………………………………………………………………………..

**2.10** Mard fis-sistema immunitarja. Jek iva, specifika

……………………………………………………………………………………………………………………………………………..

**3. Rrakkomandata ukoll:**

**3.1**  tqala? **3.1.1** Jek iva, kemm gimghat? ..................

**3.2**  Residenti fi djar tal-anzjani

**3.3** Xol fil-qasam tas-sahha? **3.3.1** Jekk iva, specifika………………………………………....

**3.4** Disabilita? **3.4.1** Jekk iva, specifika ..……………….…………………………………………….

**3.5** Kuntatt ma xi persuni bxi wahda min dawn imsemmija

**Informazzjoni genarali u eligibilita at-tilqim**

**4.1** Taf jekk t-tilqima kontra n-pneumococcus hijiex indikata ghalik?(pneumovax/previnar)

iva le

**4.2** Qatt qalulek biex tiehu t-tilqima kontra n-pneumococcus? iva le

Jek iva, min **4.2.1** Hbieb/kollegi tax-xoghol

**4.2.2** Nurse

**4.2.3** Tabib tal-familja

**4.2.4**  Specjalista

**4.2.5** Media (TV/Radju/Gazzetta/Magazine)

**4.3** Qieghed tahseb biex tehodha? iva le

**4.4** Kienu ssuggerixxewlek biex tehodha qabel tlaqt mil-isptar?

**4.4.1** Tawk ricetta? iva le

**4.5** Qatt hatt t-tilqim kontra n-pneumococcus?

**Jek le , ghajlfejn?** Tista taghzel iktar minn wahda

4.5.2.1 Biza minn side effects

4.5.2.2 Insejt/ma kellix cans

4.5.2.3 Ma kontx naf li suppost nehodha

4.5.2.4 Ghax gholja wisq

4.5.2.5 Pressjoni mil-media

4.5.2.6 Ohrjan…..………………………………………….....

**Jek iva, min?** Tista taghzel iktar minn wahda

4.5.1.1 Ghax issuggerixxiha t-tabib

4.5.1.2 Iddecidejt li niprotegi lili nnifsi

4.5.1.3 Biex niprotegi lil-familja (anzjani, tfal, ecc)

4.5.1.4 Pressjoni mil-media

4.5.1.5 Ohrajn……….……………………………………………….....

**5.1** Taf jekk t-tilqima kontra l-influenza vaccine hijiex indikata ghalik? iva le

**5.2** Qatt hatt t-tilqima kontra l-influenza? Iva le

**5.3** Tehodha kull sena? Iva le

**5.4** Fakkrek xi had biex tehodha dis-sena? Iva le

Jek iva, min 5.4.1 Hbieb/kollegi tax-xoghol

5**.4.2** Nurse

**5.4.3** Tabib tal-familja

5.4.4 Specjalista

5.4.5 Media (TV/Radju/Gazzetta/Magazine)

**5.5** Hatta t-tilqima kontra l-influenza dis-sena?

**Jek le , ghajlfejn?** Tista taghzel iktar minn wahda.

5.5.2.1 Biza mis-side effects

5.5.2.2 Insejt/ma kellix cans

5.5.2.3 Ma kontx naf li suppost nehodha

5.5.2.4 Ghax gholja wisq

5.5.2.5 Pressjoni mil-media

5.5.2.6 Ohrjan…..………………………………………….....

**Jek iva, min?** Tista taghzel iktar minn wahda.

5.5.1.1 Ghax issuggerixxiha t-tabib

5.5.1.2 Iddecidejt li niprotegi lili nnifsi

5.5.1.3 Biex niprotegi lil-familja (anzjani, tfal, ecc)

5.5.1.4 Pressjoni mil-media

5.5.1.5 Ohrajn……….……………………………………………….....
